# Whole Genome Sequencing of Field Isolates Provides Robust Characterization of Genetic Diversity in *Plasmodium vivax*

**DOI:** 10.1371/journal.pntd.0001811

**Published:** 2012-09-06

**Authors:** Ernest R. Chan, Didier Menard, Peter H. David, Arsène Ratsimbasoa, Saorin Kim, Pheaktra Chim, Catherine Do, Benoit Witkowski, Odile Mercereau-Puijalon, Peter A. Zimmerman, David Serre

**Affiliations:** 1 Genomic Medicine Institute, Cleveland Clinic Lerner Research Institute, Cleveland, Ohio, United States of America; 2 Unité d'Epidémiologie Moléculaire, Institut Pasteur du Cambodge, Phnom Penh, Cambodia; 3 Unité d'lmmunologie Moléculaire des Parasites, Institut Pasteur, Paris, France; 4 Direction de la lute contre les maladies infectieuses, Ministère de la santé, du planning familial et de la protection sociale, Antananarivo, Madagascar; 5 Center for Global Health and Diseases, Case Western Reserve University, Cleveland, Ohio, United States of America; Barcelona Centre for International Health Research (CRESIB) and Institució Catalana de Recerca i Estudis Avançats (ICREA), Spain

## Abstract

**Background:**

An estimated 2.85 billion people live at risk of *Plasmodium vivax* transmission. In endemic countries vivax malaria causes significant morbidity and its mortality is becoming more widely appreciated, drug-resistant strains are increasing in prevalence, and an increasing number of reports indicate that *P. vivax* is capable of breaking through the Duffy-negative barrier long considered to confer resistance to blood stage infection. Absence of robust *in vitro* propagation limits our understanding of fundamental aspects of the parasite's biology, including the determinants of its dormant hypnozoite phase, its virulence and drug susceptibility, and the molecular mechanisms underlying red blood cell invasion.

**Methodology/Principal Findings:**

Here, we report results from whole genome sequencing of five *P. vivax* isolates obtained from Malagasy and Cambodian patients, and of the monkey-adapted Belem strain. We obtained an average 70–400 X coverage of each genome, resulting in more than 93% of the Sal I reference sequence covered by 20 reads or more. Our study identifies more than 80,000 SNPs distributed throughout the genome which will allow designing association studies and population surveys. Analysis of the genome-wide genetic diversity in *P. vivax* also reveals considerable allele sharing among isolates from different continents. This observation could be consistent with a high level of gene flow among parasite strains distributed throughout the world.

**Conclusions:**

Our study shows that it is feasible to perform whole genome sequencing of *P. vivax* field isolates and rigorously characterize the genetic diversity of this parasite. The catalogue of polymorphisms generated here will enable large-scale genotyping studies and contribute to a better understanding of *P. vivax* traits such as drug resistance or erythrocyte invasion, partially circumventing the lack of laboratory culture that has hampered vivax research for years.

## Author Summary

*Plasmodium vivax* is the most frequently transmitted and widely distributed cause of malaria in the world. Each year *P. vivax* is responsible for approximately 250 million clinical cases of malaria and its global economic burden, placed largely on the poor, has been estimated to exceed US$1.4 billion. In contrast to *P. falciparum*, *P. vivax* cannot be propagated in continuous *in vitro* culture and this limits our understanding of the parasite’s biology. In this study, we sequenced the entire genome of five *P. vivax* isolates directly from blood samples of infected patients. Our data indicated that each patient was infected with multiple *P. vivax* strains. We also identified more than 80,000 DNA polymorphisms distributed throughout the genome that will enable future studies of the *P. vivax* population and association mapping studies. Our study illustrates the potential of genomic studies for better understanding *P. vivax* biology and how the parasite successfully evades malaria elimination efforts worldwide.

## Introduction

*Plasmodium vivax* is the most widely distributed human malaria species and causes more illness than *P. falciparum* in many regions [1]. Its global public health burden is estimated to be US$1.4 to 4.0 billion [2]. Even in areas of low transmission, up to 20% of the population can have a symptomatic infection each year, with a cumulative experience of 10–30 episodes of malaria during a lifetime [3]. Research on *P. vivax* is complicated by our inability to propagate the parasite in continuous *in vitro* cell cultures [4]. This limits our ability to perform genetic crosses, to conduct *in vitro* functional assays on anti-malarial drug susceptibility or invasion mechanisms, and RNA-based investigations. One alternative to understand phenotypic variations in *P. vivax* is to rely on purely genetic approaches and to statistically link genetic markers to traits of interests using linkage disequilibrium mapping. A first step for developing genetic studies in *P. vivax* was achieved in 2008 with the completion of the reference genome sequence [5] generated from the Sal I strain. This strain originated from a patient infected in El Salvador in 1972 and was propagated through infections of Aotus monkeys [5], [6]. A second milestone was cleared in 2010 with the first *P. vivax* genome sequenced directly from an infected patient [7], demonstrating that it was possible to sequence *P. vivax* field isolates.

Currently, both *P. vivax* genome sequences have been generated from Central/South American parasites [5], [7]. While this is an important region of endemicity, where *P. vivax* consistently predominates in prevalence over *P. falciparum* [8], these genomes only captured genetic diversity in a subset of the geographical range of *P. vivax*, and only its most recent expansion [9]. To expand our understanding of genetic diversity in *P. vivax*, we have sequenced the genome of three field isolates from Cambodia where the parasite diversity is significantly different than in Central/South America and in closer proximity to its geographic origin [9], [10]. We have also sequenced two field isolates from Madagascar where we have recently identified *P. vivax* strains capable of infecting Duffy-negative erythrocytes [11]. In addition, we have included another South American parasite, the monkey-adapted Belem strain [12], and re-sequenced the Sal I strain [5] to rigorously assess the reliability of next generation whole genome sequencing for characterizing DNA polymorphisms. Continuing advances in high-throughput sequencing technologies allowed us to generate high sequence coverage of these genomes, which circumvents most of the problems raised earlier [7] and provides reliable identification of single nucleotide polymorphisms (SNPs).

## Materials and Methods

### Ethics statement

This study was conducted according to the principles expressed in the Declaration of Helsinki. Patient samples were obtained as part of on-going studies in accordance with human studies protocols IRB N°035-CE/MINSAN (Comité d'Ethique du Ministère de la Santé de Madagascar, June 30th 2010) and IRB N°160 NECHR (National Ethics Committee for Health Research – Cambodia, October 28th 2010). All patients provided written informed consent for the collection of samples and subsequent analysis.

### Samples

We collected blood samples from two Malagasy (M08 and M19) and three Cambodian patients (C08, C15 and C127). For each patient, we processed 5 ml of fresh blood collected in EDTA vacutainers through two consecutive CF11-packed columns to remove leukocytes and platelets. We extracted parasite DNA directly from 200 µl of the remaining red blood cell fraction using DNeasy purification kit (Qiagen). For all samples, we confirmed *P. vivax* mono-species infection by *Plasmodium* species PCR-based diagnosis [13]. We also analyzed DNA extracted from the monkey-adapted Belem and Sal I strains of *P. vivax* (**Text S1**).

### Library preparation and genome sequencing

For each sample, we sheared 144–518 ng of DNA into 250–300 bp fragments using a Covaris S2 instrument and used the fragmented DNA molecules to prepare sequencing libraries according to the Illumina protocol for genomic DNA. Briefly, after end repair and A-tailing we ligated Illumina paired-end adapters to the ends of the fragmented DNA molecules and selected fragments of 300 bp (i.e. *P. vivax* fragment size of ∼250 bp) using an E-gel (Invitrogen). We then amplified the final products using 12 cycles of PCR and verified the quality and quantity of the libraries by Agilent BioAnalyzer and qPCR using the Illumina primers. We sequenced each library on one lane of an Illumina HiSeq 2000 and generated between 79 and 230 million paired-end reads of 100 bp.

We mapped all reads to the human (UCSC build hg18, [14]) and the *P. vivax* Sal I strain [5] reference genome sequences. We used the program bwa [15] to independently map each end of all read pairs. We considered as correctly mapped only reads mapped to a unique genomic location with i) less than 3 mismatches in the first 28 bases, ii) 5 or less mismatches in the 100 bp sequence and iii) at most one insertion or deletion. Only read pairs for which both ends fulfilled these criteria were included for further analyses (**Table 1**). We identified read pairs that mapped to the exact same positions (and could represent molecules amplified during the library preparation) and randomly discarded all but one pair.

**Table 1:** Sequencing and mapping summary statistics for all samples included in this study.

	C08	C15	C127	M08	M19	BELEM	SAL-I
**Sequencing and Mapping**							
# Read Pairs	231,291,984	79,414,201	211,061,945	215,643,747	85,703,544	81,446,663	215,743,944
Mapped on Human	108,735,752	46,106,026	10,366,679	117,596,401	19,186,018	92,739	3,779,857
*% mapped on human*	*47.01%*	*58.06%*	*4.91%*	*54.53%*	*22.39%*	*0.11%*	*1.75%*
Mapped on *P. vivax*	34,614,679	13,158,959	97,602,277	43,936,074	39,416,672	57,900,412	2,842,699
*% mapped on P. vivax*	*14.97%*	*16.57%*	*46.24%*	*20.37%*	*45.99%*	*71.09%*	*1.32%*
Uniquely mapped on *P. vivax*	34,479,727	13,103,714	97,259,556	43,766,410	39,260,329	57,629,725	2,831,443
*% unique*	*14.91%*	*16.50%*	*46.08%*	*20.30%*	*45.81%*	*70.76%*	*1.31%*
After removal of duplicated read pairs	13,277,182	9,595,428	54,955,137	29,260,300	15,536,615	54,422,730	2,760,241
*% duplication*	*61.49%*	*26.77%*	*43.50%*	*33.14%*	*60.43%*	*5.56%*	*2.51%*
**Coverage**							
Average nucleotide coverage	102 X	70 X	407 X	218 X	117 X	418 X	20 X
% genome covered> = 20 X	93.22%	93.50%	97.40%	95.36%	97.02%	95.73%	51.43%
# Genes covered*> = 20 X	4,229	4,797	4,905	4,618	4,911	4,908	401

The table indicates for each sample sequenced in this study, the number of read pairs generated, the numbers and percentage mapped to the human and *P. vivax* reference genomes, as well as for read pairs mapping to *P. vivax*, the numbers of reads mapped to a unique location and the number of reads remaining after removal of molecules potentially amplified during the library preparation. The bottom part shows the final coverage summaries for each sample.

* at least 90% of the protein coding region sequenced at 20 X(n = 5,050).

### Identification of Single Nucleotide Polymorphisms

To identify SNPs, we focused on nucleotide positions covered by at least 20 reads with a quality score greater than 30. Since SNP identification is complicated in regions of high homology, we excluded from our analysis possible paralogous sequences (see **Text S1** for details). In addition, we considered only read pairs that mapped in head-to-head configuration and within 1,000 bp of each other. We identified consistent mismatches between reads generated from a given sample and the reference genome sequence using the samtool mpileup [16] with the extended base alignment quality computation. Finally, we only considered a position variable in a given sample if at least 10% of the reads differed at this position from the reference nucleotide (i.e. Reference Allele Frequency [RAF] <90%). We characterized the function of each DNA polymorphism identified using the Sal I gene annotation downloaded from Ensembl. We used perl scripts to annotate whether each polymorphism occurred in an intergenic region or a protein-coding gene and for the latter, whether it resulted in an amino acid change or a premature termination.

### Duffy Binding Protein sequence analysis

We reconstructed individual haplotypes (i.e. the haploid DNA sequence of each *P. vivax* strain present in a sample) from the short read sequences mapped at the Duffy Binding Protein (DBP) locus. We retrieved, for each sample, all reads mapping to 1 kb upstream and downstream of the DBP gene (PVX_110810, chr6:384,498–390,259) and recorded all co-occurrences of consecutive alleles on read pairs: since read pairs are generated from the sequencing of the ends of individual DNA molecules, alleles observed on the same read pair are carried by the same haplotype. For this analysis we focused on common haplotypes and only analyzed polymorphisms with a minor allele frequency of at least 5% in the sample studied (i.e. only sites variable within a given sample are considered). We inferred the haplotype using direct information when available, or allele frequency. The final haplotype sequences were generated by substituting, in the Sal I reference sequence, the variable positions (i.e. the haplotype polymorphisms inferred as well as the alleles at positions where all strains of a sample differed from the reference sequence, i.e. RAF <5%). For the Belem strain none of the mismatches reached an allele frequency of 5%, consistent with a single strain being present in the infected monkey.

For five of the samples sequenced in this study, we amplified the Duffy Binding Protein region II from genomic DNA and, after cloning the PCR products, sequenced 12–91 clones per sample by traditional Sanger technology.

### Determination of the dominant haplotype and allele sharing

We reconstructed the haploid DNA sequence of the major strain across the entire genome using, at each nucleotide position, the most frequently observed allele. Analytical and re-sampling approaches (see **Text S1** for details) showed that this method performs well when one strain represents more than 80% of the parasite DNA (using a minimum coverage of 20 X).

We analyzed allele sharing across samples by comparing the haploid DNA sequences of C08, C127, M15, Sal I and Belem. For each annotated protein coding DNA sequence, we calculated the number of nucleotide differences between each pair of samples and determined which haplotypes were closest (i.e. lowest number of differences).

### Genome-wide search for signals of local adaptation

We looked for signals of local selection across the entire genome by searching for nucleotide positions where one allele was fixed in one population and the other allele fixed in the other populations. To deal with multiple infections, we considered that all strains in one sample had the same allele if >90% of the reads carried this allele (we used 90% instead of 100% to account for possible sequencing errors).

## Results and Discussion

### Genome sequencing of field isolates and monkey-adapted *Plasmodium vivax* strains

Studies of malaria parasites obtained from blood samples of infected patients are complicated by the presence of human genomic DNA [7]: due to the difference in genome size, if only one leukocyte is present per 10 parasite cells, more than 95% of the extracted DNA will be of human origin. This is particularly problematic in studying *P. vivax* as its parasitemia is typically less than 10,000 parasites per µl of blood.

Here, we analyzed blood samples from five malaria patients (**Table S1**), two from Madagascar (M08 and M19) and three from Cambodia (C08, C15 and C127). We processed blood samples through cellulose columns to remove leukocytes and platelets [17], [18] and extracted DNA from the red blood cell fraction. In addition, we analyzed *P. vivax* DNA from the monkey-adapted Belem strain (**Text S1**) as well as from the Sal I strain used for generating the *P. vivax* reference genome sequence [5]. After library preparation, we sequenced each sample on one lane of an Illumina HiSeq 2000 to generate between 79 and 231 million paired-end reads of 100 bp (**Table 1**). We mapped all reads to the *P. vivax* (Sal I, [5]) and human [14] reference genome sequences.

The Belem strain showed 71.09% of the reads mapping to the *P. vivax* genome (**Table 1**), resulting from the extensive effort to remove leukocytes from the blood sample (**Text S1**). By contrast, only 1.32% of the reads generated from Sal I DNA mapped to the *P. vivax* genome. This figure can be explained by the absence of leukocyte depletion prior to DNA extraction of the Sal I sample and illustrates the benefits of processing fresh blood samples on cellulose columns to remove host DNA. Despite its relatively low coverage (20 X), we included Sal I in our analyses since comparison with the reference genome sequence generated from the same strain provided an opportunity to estimate the false positive rate of our SNP calling approach.

**Figure 1:**
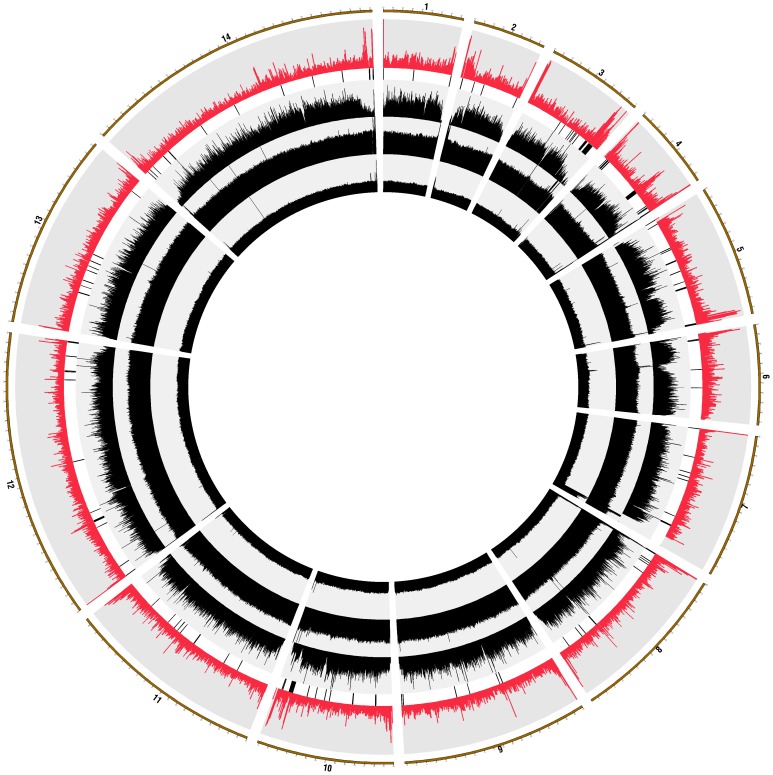
Overview of the genetic diversity in *P. vivax* genomes. The 14 *P. vivax* chromosomes are organized circularly. The red histograms show the number of SNPs per 5 kb (varying from 0 to 100); the vertical black bars indicate regions masked for SNP analysis as putative paralogous sequences. The concentric black histograms represent, from outside to inside, the sequence coverage for Belem, C127 and M08 respectively.

For the field isolates, a variable proportion of reads (4.91%–58.06%) could be mapped to the human genome (**Table 1**), consistent with incomplete leukocyte depletion from the blood samples. Despite this residual human DNA contamination and stringent quality controls, which eliminated between 27 and 62% of the reads mapped to *P. vivax* (**Table 1**), the amount of DNA sequence generated provided high coverage of the *P. vivax* genomes (between 70 X and 407 X, **Table 1** and **Figure 1**). However, the average genome coverage does not accurately represent the quality of the sequencing data. Dharia et al. [7] sequenced the first *P. vivax* field isolate at an average genome coverage of 30 X. This 30 X coverage translated into 24.89% of the Sal I nucleotides being covered by more than 20 high-quality reads, and only 3% of the genes (158 out of the 5,050 annotated genes in the Sal I genome) having more than 90% of their coding region sequenced at this coverage. By contrast, owing to continuing advances in massively parallel sequencing, the Malagasy and Cambodian samples analyzed here had at least 93% of their genome sequenced by 20 reads or more, and between 84 and 97% of the genes covered (**Table 1**).

### Identification of Single Nucleotide Polymorphisms

**Figure 2:**
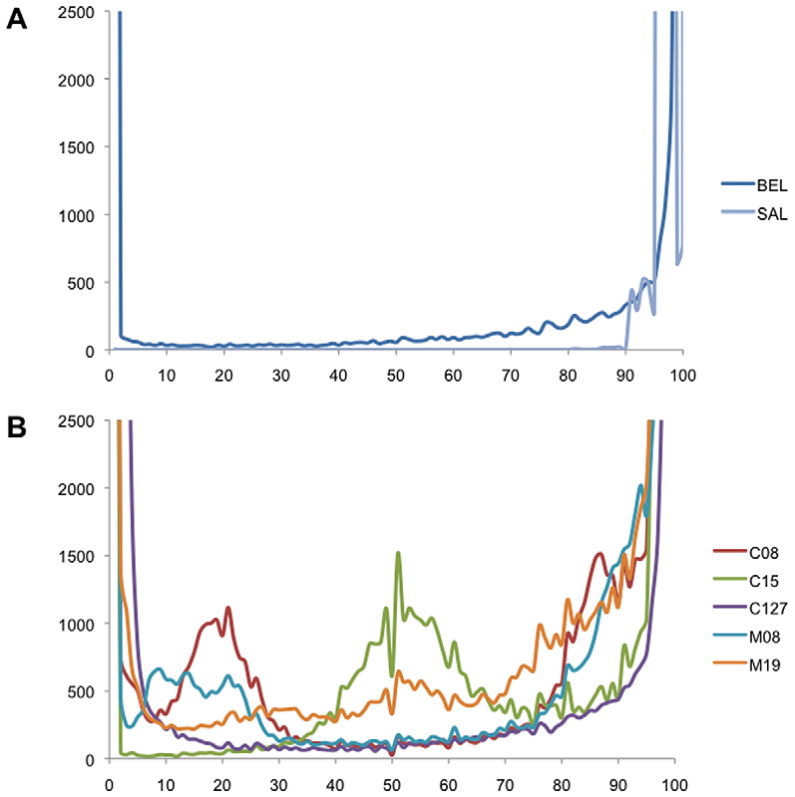
Reference Allele Frequency in (A) monkey-adapted strains and (B) field isolates. Each variable nucleotide position observed in a sample is displayed according to the proportion of reads carrying the reference allele (x-axis). The y-axis shows the number of variable positions with a given RAF.

To determine whether we could use whole genome sequence data to identify DNA polymorphisms, we first compared the reads generated from Sal I DNA to the reference genome previously sequenced from the same strain [5]. Out of ∼11.6 million nucleotide positions covered by 20 reads or more, only 121 nucleotides differed from the reference nucleotides in more than 10% of the reads covering those positions, and none were supported by more than 90% of the reads (see **Figure 2A** and **Text S1**). These results highlighted both the high quality of the assembled *P. vivax* reference genome sequence and the suitability of high coverage genome sequence data for identifying SNPs with low false positive rates.

For all further analyses, we focused on positions of the Sal I reference genome that were covered by at least 20 reads in the Belem strain and each field isolate (i.e., all samples we sequenced excluding the low coverage Sal I). We also excluded from our analyses potentially paralogous sequences that could generate spurious SNP calls (**Text S1**). Overall, 19,533,315 nucleotides (86.35% of the Sal I reference genome) were included in the SNP analysis.

For each sample, we recorded the percentage of reads differing from the reference nucleotide at each position. For the monkey-adapted *P. vivax* Belem strain, at any given nucleotide position, all reads carried the reference allele (i.e. 100% Reference Allele Frequency [RAF]) or all reads differed from the Sal I reference (0% RAF) (**Figure 2A**). This is consistent with *P. vivax* being haploid in the human/monkey host and with the presence of a single strain in the Saimiri monkey. Note that the RAF at some positions differed slightly from 0 or 100% (typically by less than 5%) due to sequencing errors.

The distribution of RAF was strikingly different for the *P. vivax* field isolates: in these samples, we consistently observed two alleles at many positions (**Figure 2B**). This pattern suggested that multiple strains of *P. vivax* were present in each patient blood sample. For example, a SNP with an RAF of 20% (as it was frequently observed in C08) could occur if two strains of *P. vivax* were present in the patient blood and the major strain (accounting for 80% of the parasites) differed from the reference allele at this position while the minor strain (making up the remaining 20% of the parasites) carried the Sal I reference nucleotide. The peaks at 0% RAF in **Figure 2B** represent positions where all strains present in a sample differed from the Sal I reference allele.

We independently validated a subset of the SNPs by cloning and Sanger sequencing the Duffy binding protein region II (DBPII) for five of our samples. The Sanger sequencing results were consistent with whole genome sequence findings and validated 17 out of the 17 SNPs identified by Illumina sequencing in this region (**Table S2**). In addition, analysis of the cloned sequences revealed distinct haplotypes amplified from a single blood sample, confirming the presence of multiple strains in two of the samples (**Figure S1**).

Overall, we identified 80,657 nucleotide positions where at least 10% of the reads differed from the reference sequence in one or more of the samples. The 80,657 SNPs were distributed throughout the genome with an average of 4.13 SNPs per kb. However, there was extensive variation in SNP density among genomic regions (**Figure 1**). Most notably, the extent of genetic diversity was highly dependent on the gene context: intergenic regions showed a much higher diversity than coding regions (6.98 vs. 2.96 SNPs per kb, respectively) with intronic sequences harboring an intermediate level of diversity (4.29 SNPs per kb). This observation was similar to the results of a study of 100 kb of contiguous DNA sequence [19] and consistent with purifying selection maintaining the DNA sequence at most genes in the *P. vivax* genome by removing deleterious mutations. 48,224 SNPs occurred in intergenic regions. SNPs in annotated protein coding regions included 13,203 synonymous polymorphisms (sSNPs, 16.37% of all SNPs), 19,191 non-synonymous polymorphisms (nsSNPs, 23.79%) and 39 substitutions (0.05%) introducing an early stop codon. We only observed 1.5-fold more nsSNPs than sSNPS, while based on the composition of the *P. vivax* genome we would expect by chance ∼4-fold more nsSNPs. This observation also suggested that the evolution of most protein-coding sequences in *P. vivax* genome is driven by purifying selection.

### Reconstruction of haplotype sequences of individual *P. vivax* parasites

**Figure 3:**
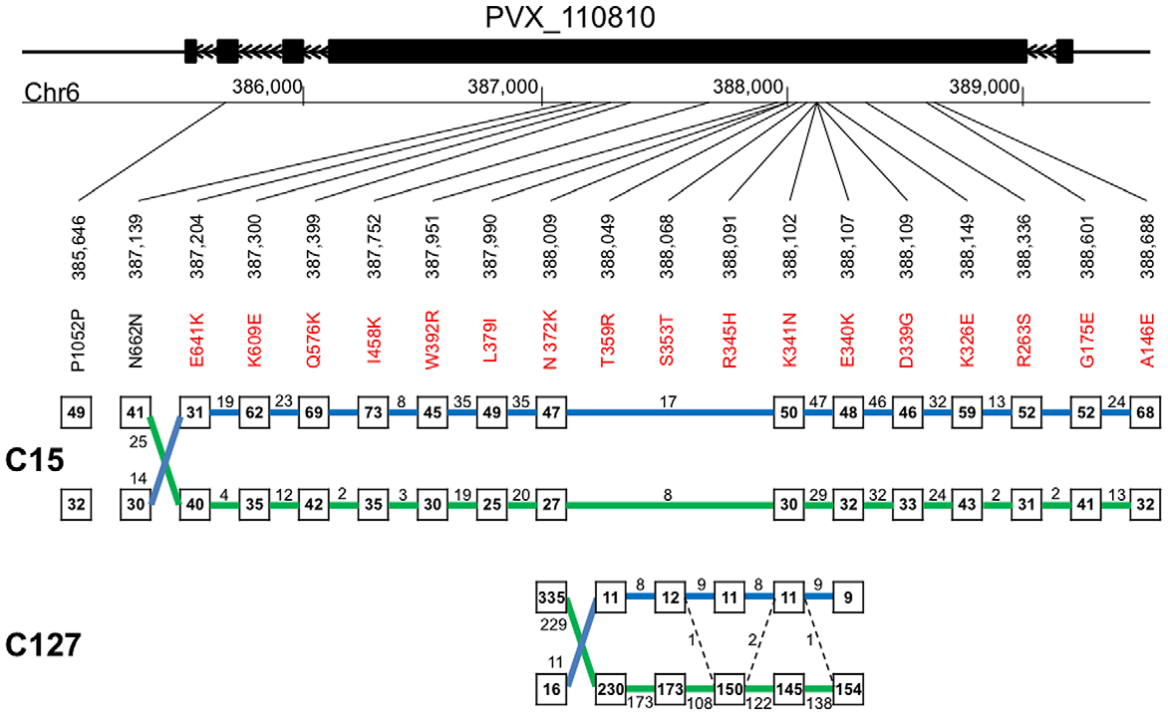
Haplotype reconstruction at the Duffy binding protein locus for C15 and C127. The figure displays, from top to bottom, the Duffy binding protein structure, its chromosomal location, the polymorphic positions in C15 and C127 (non-synonymous polymorphisms are shown in red) and the haplotype reconstruction for C15 and C127. The numbers in boxes represent for each sample the number of reads carrying the reference (top row) or alternative (bottom row) alleles, while the numbers above the lines joining boxes indicate the number of reads carrying two consecutive alleles. The reconstructed haplotypes are shown by the green and blue lines. Note that for C127, a few reads support the presence of a third haplotype (dashed lines).

We attempted to assign allelic variants observed within a sample to individual *P. vivax* parasites for the Duffy binding protein locus. For each sample, we recorded the co-occurrence of consecutive alleles on individual read pairs: since read pairs were generated by sequencing the ends of single DNA molecules, alleles observed on the same read pair were carried by the same parasite. Using this procedure, we were able to reconstruct haplotypes for the most prevalent strain for all samples as well as for a second strain for the C15, C127 and M08 field isolates (**Figure 3** and **Figure S2**). The inferred haplotypes were identical to the consensus DBPII sequences generated by cloning and Sanger sequencing from the same samples (**Figure S1**), validating our haplotype reconstruction approach.

The haplotypes inferred from the same patient blood sample were not closely related to each other and represented unrelated *P. vivax* parasites. It is important to note here that while sequencing data allows identifying genetically distinct parasites, it does not differentiate related parasites derived from parental strains by mutations or recombination (see e.g. [20]).

Haplotype reconstruction, when several strains are present in a single sample, depends on several factors, including the SNP density and the extent of linkage disequilibrium. This currently hampers extending our approach to the entire genome. However, the analysis of Duffy binding protein haplotypes was consistent with the distribution of allele frequencies shown on **Figure 2** and indicated that, in all field samples, 2–4 strains contributed to more than 95% of the *P. vivax* DNA (as opposed to a scenario where dozen of strains would be equally abundant in a patient). Two strains were equally abundant in C15, while for M19 three strains dominated (with roughly 50%, 25% and 25% frequency). In three samples, M15, C08 and C127, one single strain largely dominated all others and represented, respectively, 80%, 80% and >90% of the parasites present. Given the high sequence coverage generated here, for these samples, the most frequently observed allele at each nucleotide position was very likely carried by the dominant strain (see **Text S1** for details). We therefore inferred for these three samples the haploid sequence of the dominant strain for the entire genome by considering the major allele at each variable position. Combined with the single strain sequences of Belem and Sal I, these sequences provided five haploid genome sequences for *P. vivax* from three continents.

### Preliminary assessment of the global genetic diversity in *P. vivax*

Studies of the global *P. vivax* genetic diversity have been limited by the lack of informative markers and essentially based on a few loci (e.g. the mitochondrial DNA [9], [21] and the Duffy binding protein [22]). This has greatly limited our understanding of the *P. vivax* population structure and history since diversity at these loci may be influenced by natural selection. The five haploid genome sequences generated here provided an opportunity to preliminarily assess the global genetic diversity of *P. vivax*.

Identification of likely neutral sequences in the *P. vivax* genome is complicated by its gene density: less than half of the genome sequence is intergenic and there are few long stretches (e.g. ≥10 kb) of DNA sequences without annotated genes. We therefore focused on the analysis of four-fold degenerate sites (i.e., nucleotide positions where substitutions do not change the amino acid sequence) that are less likely to be directly affected by natural selection. Among 98,393 four-fold degenerate sites sequenced, we observed 2,193 variable sites, including 1,769 sites that differed in only one sample. This represented a significant excess of singletons compared to the number expected under a neutral model of a random mating population of constant size and could indicate that *P. vivax* population has recently expanded in size, or alternatively, that the parasite population is heterogeneous and composed of many sub-populations.

**Figure 4:**
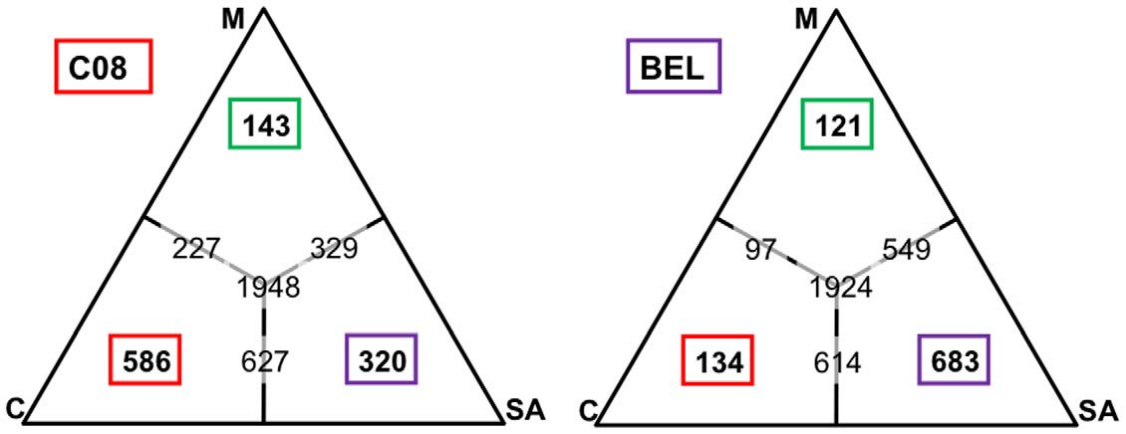
Allele sharing among *P. vivax* parasites. The ternary plot shows the sample carrying the closest haplotype (i.e. the haploid sequence with the smallest number of nucleotide differences) to C08 (left) and Belem (right) for all annotated genes. The number in the red box indicates the number of genes for which the closest sequence is the C127 haplotype, while the green and purple boxes indicate the numbers of genes for which the closest sequence are, respectively, the M15 and Sal I haplotypes. The numbers of the edges represent genes for which two or more haplotypes are equally distant from the sample considered.

Consistent with previous reports [22], our analysis of Duffy binding protein sequences showed a star-like phylogeny with no apparent geographic stratification (**Figure S3**). This pattern could reflect the actual structure of the *P. vivax* population or simply indicate the action of natural selection on the DBP gene. To further investigate population structure in *P. vivax*, we compared the five haploid sequences and determined, for each annotated gene, the geographical origin of the closest haplotype to a given sample (similar to the nearest neighbor approach described in [23]). Our results showed that, while strains from the same location tended to be more similar to each other than to a strain from a different continent, there was considerable allele sharing across continents (**Figure 4**). For example, the haplotype sequence for the Cambodian sample C08 was most similar to the Cambodian C127 haplotype for 586 genes but most similar to the Malagasy M15 or South American Belem haplotypes at 463 genes. Consistent with this observation, a tree reconstructed using the total number of nucleotide differences among whole genome haploid sequences showed that strains from a same continent clustered together but with very long external branches (**Figure S4**), indicating that most diversity is observed among samples rather than between geographical locations.

Previous studies based on mitochondrial DNA [24] and microsatellites [25] have also highlighted that similar haplotypes are often shared across continents (but see also [26]). This observation of extensive allele sharing across continents is unexpected as we may have expected to observe consequences of local adaptation, and therefore greater population differentiation, as *P. vivax* spread across the world and encountered new environments (e.g., different mosquito species, different host's immune response). We screened the *P. vivax* genome for evidence of adaptive selection by looking for SNPs for which one allele was fixed in all parasites from one geographical area while the other allele was fixed in all other parasites. For comparison, this is the situation at the Duffy locus in humans where Duffy-negativity is fixed in Sub-Saharan Africans and absent in non-African populations. Among the 80,657 SNPs identified, we only observed 96 SNPs with such dramatic allele frequency differences (not statistically different from the number expected by chance due to the small number of samples analyzed). In addition, while all three Cambodian-specific alleles occurred in close proximity (within 20 bp from each other), the 92 Malagasy-specific alleles were distributed across the 14 chromosomes suggesting that chance rather than natural selection was responsible for these results. This analysis was consistent with our observation of allele sharing across continents and suggested that *P. vivax* population is not highly differentiated.

In conclusion, we showed that continuing advances in sequencing technology allow the robust characterization of genetic diversity in *P. vivax* genomes. The SNPs identified here will be valuable for vivax malaria research to design population studies (e.g. studying the diversity of *P. vivax* in one region) and to identify the genetic basis of disease-related traits by association studies. In this regard, it is important to note that we identified multiple parasites in each patient blood sample analyzed, which will complicate these studies and will need to be rigorously accounted for.

Finally, our analysis of *P.vivax* genomes from three continents revealed allele sharing across continents and little evidence of local adaptations. While our analysis includes, for the first time, genetic diversity estimates across the entire genome, the number of samples analyzed here is limited. We conducted population genetic analyses using approaches robust to small sample sizes but our results will need to be confirmed as more genome sequences become available for this parasite. One possible explanation for our observations is that the *P. vivax* population originated recently and dispersed rapidly across the world without major loss of diversity or much influence of natural selection. Alternatively, allele sharing could be due to continuous gene flow in the present *P. vivax* population: *P. vivax* is now a cosmopolitan parasite that can be easily spread throughout the world by way of dormant hypnozoites. If this second hypothesis is true, it holds bleak prospects for vivax malaria elimination: with high level of gene flow, genetic polymorphisms conferring drug resistance [27], [28] or novel invasion mechanisms [11] could spread across the world and further complicate control strategies.

## Supporting Information

Text S1 **Additional materials and methods.**
(DOC)

Figure S1 **Neighbor-joining tree showing the relationships among PvDBPII sequences obtained directly by cloning and Sanger sequencing (full circles) and inferred haplotypes from whole genome sequencing (empty circles) for 5 samples.** Sequences from different samples are represented by different colors. Clustering of M19 and M08 sequences on distinct branches reveals the presence of multiple strains in these samples (with respectively 4 and 2 distinct strains).
(TIF)

Figure S2 **DBP haplotype for all samples.** See legend of **Figure 3** for details.
(TIF)

Figure S3 **Neighbor-Joining tree reconstructed using the inferred DBP haplotypes (red arrows) and Sanger sequences downloaded from NCBI.**
(TIF)

Figure S4 **Tree reconstructed using the total number of nucleotide differences among haploid genomes.**
(TIF)

Table S1 **Characteristics of the patient samples included in the study.**
(PDF)

Table S2 **Comparisons of the SNPs identified by whole genome sequencing (WGS) and cloning/Sanger sequencing for the DBPII region.** **Table S2A** shows, for each sample and at each polymorphic position of the DBPII, the number of WGS reads supporting the reference/alternative alleles. The table also indicates the number of clones supporting each consensus haplotype sequence (only nucleotides differing from the reference sequence are indicated). **Table S2B** displays the resulting reference allele frequency at each position for each sample.
(PDF)
