# Supplementary material for: Whole Genome Sequencing of Field Isolates Provides Robust Characterization of Genetic Diversity in Plasmodium vivax
Source: PLoS Negl Trop Dis. 2012 Sep 6;6(9):e1811. doi: 10.1371/journal.pntd.0001811 (PMC3435244; doi:10.1371/journal.pntd.0001811)
Supplement: Text S1 — Additional materials and methods. (DOC) [file pntd.0001811.s001.doc]

**Whole Genome Sequencing of Field Isolates Provides Robust Characterization of Genetic Diversity in *Plasmodium vivax***

Ernest R. Chan, Didier Menard, Peter H. David, Arsène Ratsimbasoa, Saorin Kim, Pheaktra Chim, Catherine Do, Benoit Witkowski, Odile Mercereau-Puijalon, Peter A. Zimmerman, David Serre

**Supporting Information**

**Materials and Methods**

**DNA extraction from the Belem strain of P. vivax**

The Belem strain of *Plasmodium vivax* was originally isolated from a patient in Belem (Brazil) in 1980 by Mercia de Arruda, adapted to Saimiri monkeys by Jurg Gysin and has been maintained in squirrel monkeys thereafter. Splenectomized *Saimiri sciureus guyanensis* were rendered anemic by repeated bleeding before infection with the *P. vivax* Belem parasites. When parasitemia reached 2-5% (predominantly schizonts), blood was collected by venipuncture on heparin. Blood collected from four monkeys was pooled, adjusted to 1mM ADP, filtered through glass beads (1V blood/1.25V glass beads), washed with RPMI and resuspended in 2V RPMI. Blood was then filtered through two CF11 columns equilibrated in RPMI (2V each), washed with RPMI and the red blood cells recovered by centrifugation at 2,000 rpm. The pellet was resuspended in 5 ml RPMI at ~30% hematocrit, loaded on 40/60 Percoll gradient and centrifuged at 3,500 rpm for 15 minutes at room temperature. Schizonts and trophozoites were recovered at the 40/60 interface, washed and resuspended in RPMI, 10% anti-human PBMC rabbit serum thoroughly pre-adsorbed on RBCs, incubated for 15 minutes at room temperature, filtered onto proteinA-Sepharose 6B column, and recovered by centrifugation. Finally, DNA was extracted from the RBC pellet using phenol/chloroform.

**DNA sequence differences between Sal I reads and the Sal I reference genome**

To identify nucleotides that differed between the Sal I reads and the Sal I reference genome sequence, we focused on 19,311,001 nucleotides (85.35 % of the genome) that were covered by at least 10 reads. Overall, 1,035 nucleotides differed from the reference nucleotides in more than 25% of the reads and only 145 of these mismatches were supported by more than 90% of the reads. The latter number illustrates the high quality of the *P. vivax* reference genome sequence as these few events represent a combination of recurrent sequencing errors in the next generation sequencing data, systematic errors of the mapping algorithm, possible genuine differences between the two samples of Sal I *P. vivax* (e.g. mutations that accumulated between the collection of the two samples) and sequencing errors in the reference genome (the numbers corresponding to 20 X coverage are presented in the main manuscript).

To identify putative paralogous sequences existing in the Sal I genome but collapsed into a single copy in the reference genome assembly, we screened the genome for regions that contained more mismatches than would be expected by chance (assuming that these mismatches occurred randomly). We split the Sal I reference genome sequence into 5 kb windows and calculated for each window the numbers of mismatches between Sal I reads and the reference genome sequence. We then estimated that any window containing more than four mismatches was unlikely to occur by chance (FDR < 1%) and could indicate the presence of an unannotated paralogous sequence (or a region where alignment errors happen systematically). We therefore masked these 21 windows of 5 kb for the SNP analysis. We also masked 148 regions of 5 kb where multiple paired end reads did map in the expected configuration (i.e. head to head and with 1 kb from each other) which could indicate locations of sequence rearrangements or regions where high sequence homology led to possible incorrect mapping.

**Determination of the dominant strain haplotype**

To determine whether we could use the major allele at each position to reconstruct the haploid genome sequence of the most abundant strain in samples containing multiple parasites, we used two approaches.

First, we estimated the probability of making a wrong call depending on the proportion of the dominant strain in a sample using a binomial distribution. Since we called nucleotides at any position covered by at least 20 reads, we calculated the probability of selecting, from 20 random draws, less than 10 reads from the dominant strain given its relative proportion. Note that our estimates are very conservative since i) most positions in the genome have much higher sequence coverage and ii) we assumed here that all other strains present in the sample differed at that position from the dominant strain. The probability of making a wrong inference drops rapidly as the frequency of the dominant strain increases to reach 5.6x10^-4^ when the dominant strain represents >80% of the parasites. In our analyses, we typically observed 20,000-30,000 differences between two strain’s genomes which would lead to less than 17 wrong calls across the genome if the dominant strain makes up 80% of the parasites:

| Proportion of major strain | 90% | 80% | 70% | 60% |
| --- | --- | --- | --- | --- |
| # wrong calls* | <1 | 17 | 515 | 3,826 |

*assuming 30,000 nucleotide differences between strains

Second, we assessed how well the dominant strain can be reconstructed by mixing reads from two single-strain samples. We randomly sampled reads from two monkey-adapted *P. vivax* (India VII and Brazil) sequenced at the Broad Institute to obtain an average genome coverage of 100 X. We analyzed reads mapping to 1 Mb of DNA on chromosome 14 (chr14:500,000-1,500,000) and only considered positions with a coverage > 20 X (following the approach used in our analyses). Out of 921 differences between the two monkey-adapted strains for this region, we only incorrectly called 14 positions (1.5%) when mixing the two strains 80%-20%. When mixing 70%-30%, 30 positions (3.3%) were incorrectly called (the allele of the minor strain was most frequently observed) and one position was incorrectly called due to sequencing errors. The error rate in the mixing experiment is greater than expected statistically (see above) and likely due to variations in sequence coverage along the genome: in contrast to a field-based multiple-strain infection, the sequence data for our mixing experiment was generated independently for the two strains (i.e. from DNA extraction to shearing, library prep and sequencing), which increases differences in coverage and represents a worst-case scenario.
