# Supplementary figures and images for: Whole Genome Sequencing of Field Isolates Provides Robust Characterization of Genetic Diversity in Plasmodium vivax
Source: PLoS Negl Trop Dis. 2012 Sep 6;6(9):e1811. doi: 10.1371/journal.pntd.0001811 (PMC3435244; doi:10.1371/journal.pntd.0001811)

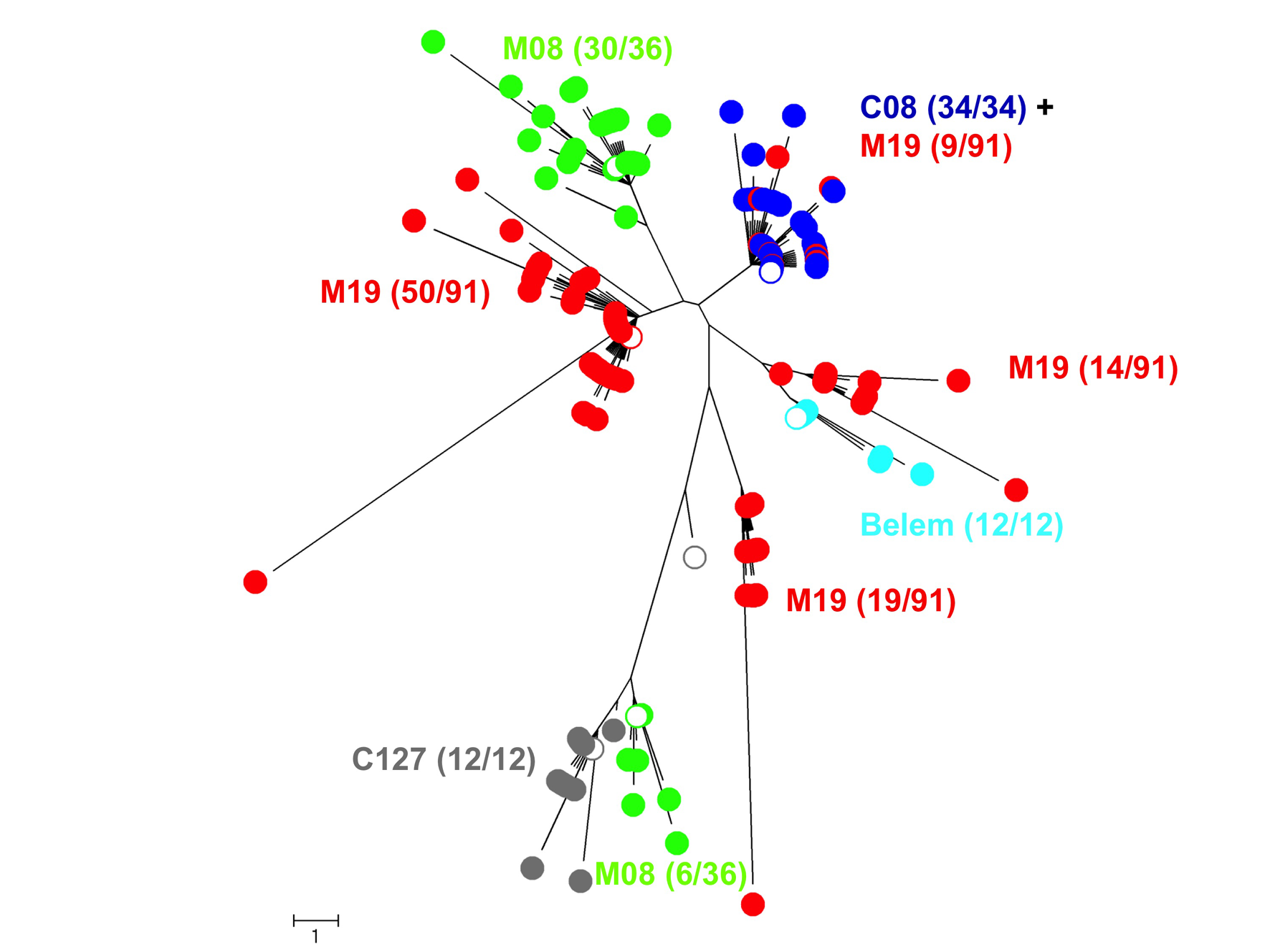

Supplement: Figure S1 — Neighbor-joining tree showing the relationships among PvDBPII sequences obtained directly by cloning and Sanger sequencing (full circles) and inferred haplotypes from whole genome sequencing (empty circles) for 5 samples. Sequences from different samples are represented by different colors. Clustering of M19 and M08 sequences on distinct branches reveals the presence of multiple strains in these samples (with respectively 4 and 2 distinct strains). (TIF) [file pntd.0001811.s002.tif]

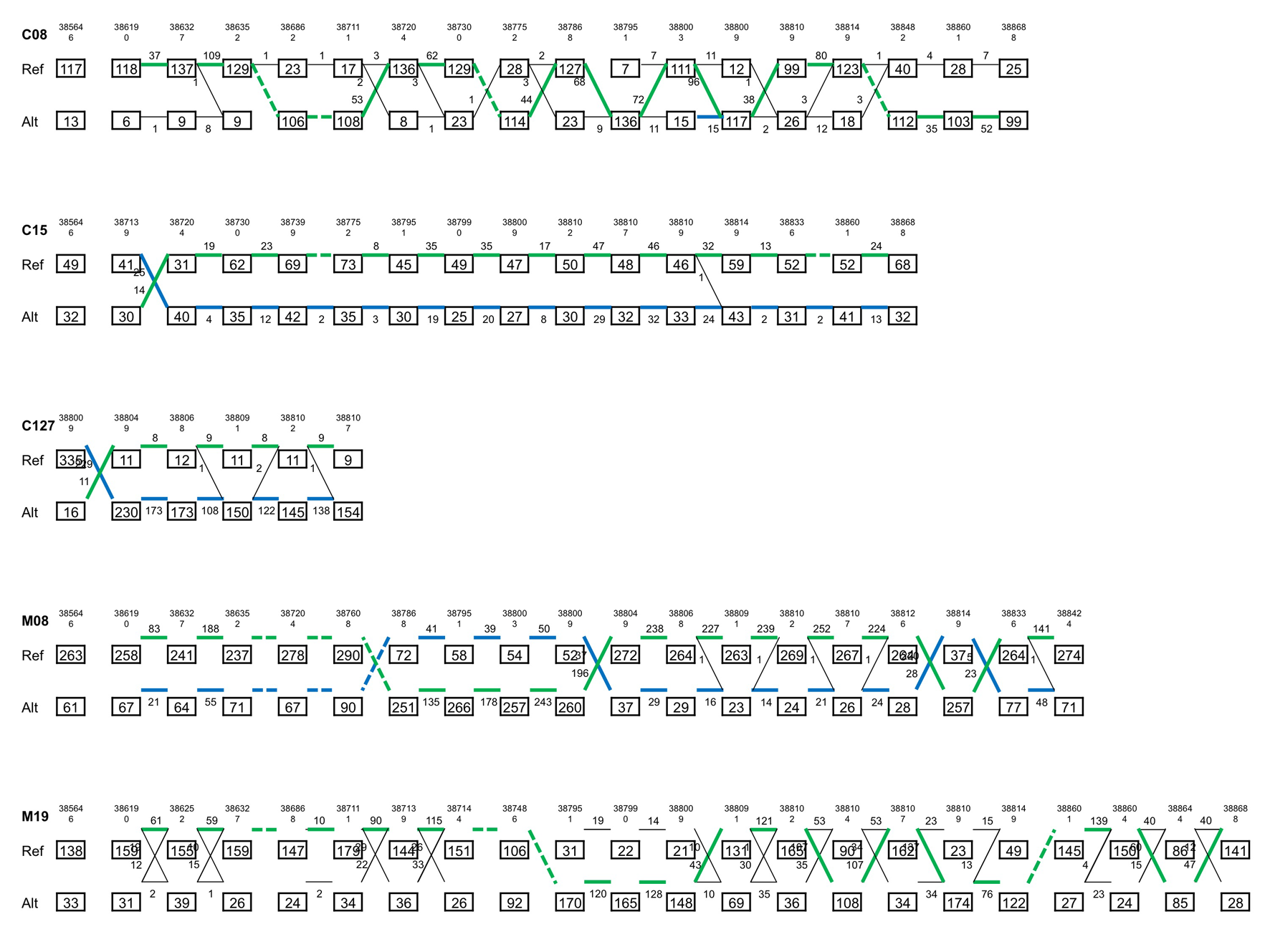

Supplement: Figure S2 — DBP haplotype for all samples. See legend of Figure 3 for details. (TIF) [file pntd.0001811.s003.tif]

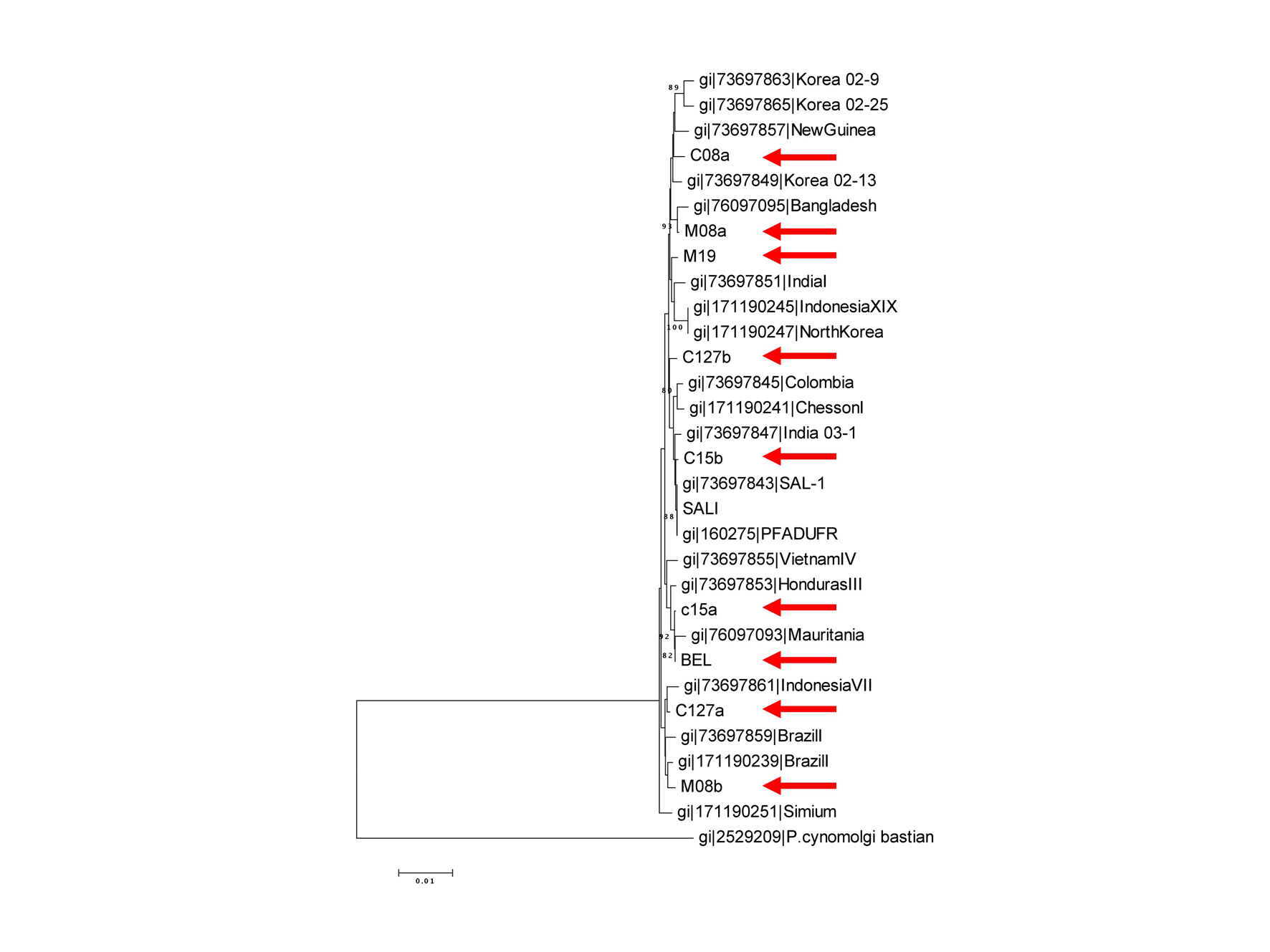

Supplement: Figure S3 — Neighbor-Joining tree reconstructed using the inferred DBP haplotypes (red arrows) and Sanger sequences downloaded from NCBI. (TIF) [file pntd.0001811.s004.tif]

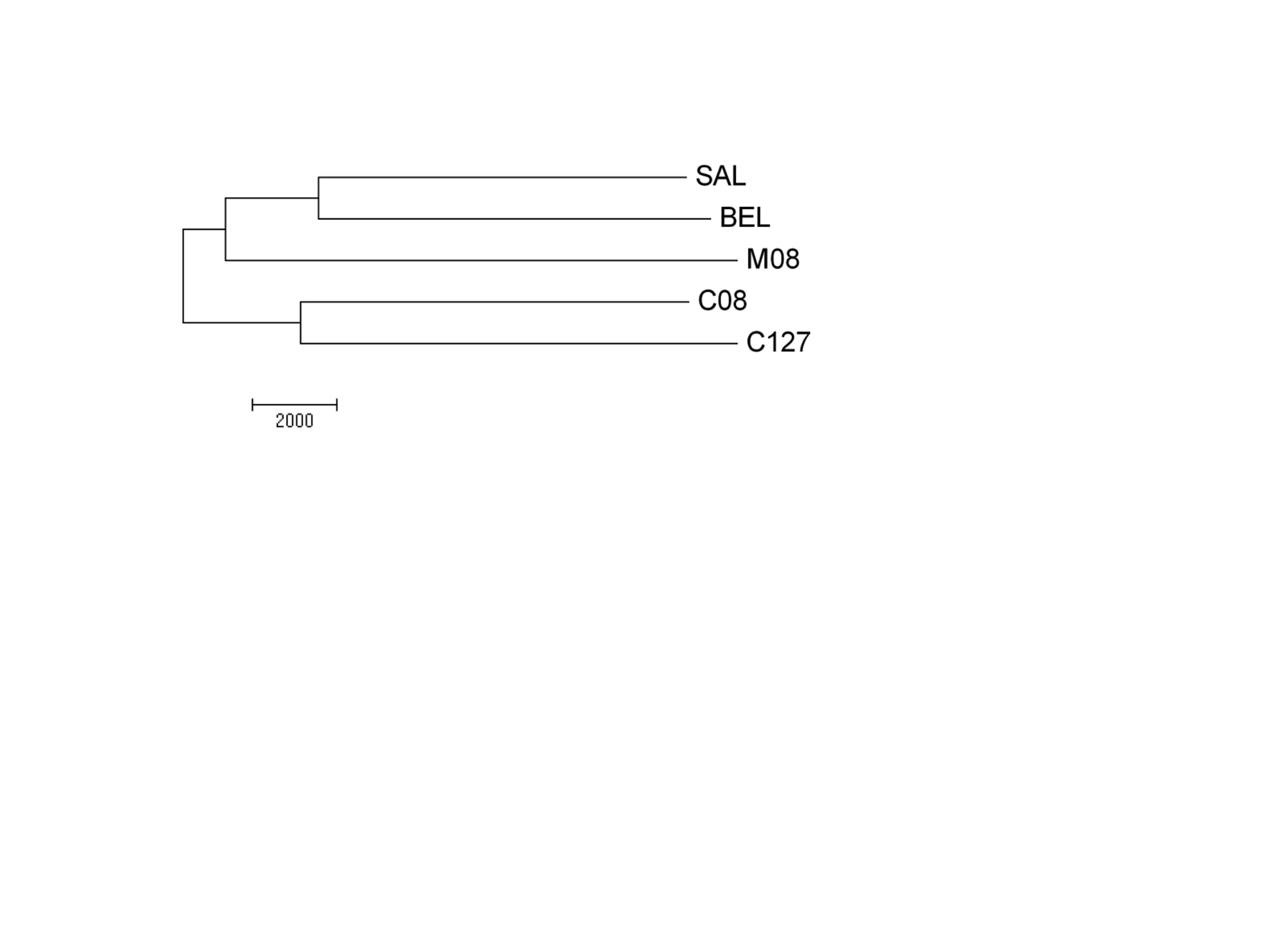

Supplement: Figure S4 — Tree reconstructed using the total number of nucleotide differences among haploid genomes. (TIF) [file pntd.0001811.s005.tif]
