# Supplementary material for: Whole Genome Sequencing of Field Isolates Provides Robust Characterization of Genetic Diversity in Plasmodium vivax
Source: PLoS Negl Trop Dis. 2012 Sep 6;6(9):e1811. doi: 10.1371/journal.pntd.0001811 (PMC3435244; doi:10.1371/journal.pntd.0001811)
Supplement: Table S1 — Characteristics of the patient samples included in the study. (PDF) [file pntd.0001811.s006.pdf]

Supplemental Table S1. Characteristics of the patient samples included in the study.

	**C08**	**C15**	**C127**	**M08**	**M19**
Origin	Cambodia	Cambodia	Cambodia	Madagascar	Madagascar
Collection Site	Pailin	Takavit	Takavit	Ampasimpotsy-V13	Ampasimpotsy-V13
Date of Collection	May 2011	January 2011	May 2011	August 2010	August 2010
Patient's age	20 years	20 years	14 years	3 years	3 years
Patient's sex	Male	Male	Male	Female	Female
Patient's temperature	37.2°C	39.0°C	40.0°C	37.6°C	37.7°C
Parasitemia (parasite/µl)	~20,000	~50,000	~20,000	~18,000	~8,000
