# Supplementary material for: Whole Genome Sequencing of Field Isolates Provides Robust Characterization of Genetic Diversity in Plasmodium vivax
Source: PLoS Negl Trop Dis. 2012 Sep 6;6(9):e1811. doi: 10.1371/journal.pntd.0001811 (PMC3435244; doi:10.1371/journal.pntd.0001811)
Supplement: Table S2 — Comparisons of the SNPs identified by whole genome sequencing (WGS) and cloning/Sanger sequencing for the DBPII region. Table S2A shows, for each sample and at each polymorphic position of the DBPII, the number of WGS reads supporting the reference/alternative alleles. The table also indicates the number of clones supporting each consensus haplotype sequence (only nucleotides differing from the reference sequence are indicated). Table S2B displays the resulting reference allele frequency at each position for each sample. (PDF) [file pntd.0001811.s007.pdf]

Supplemental Table S2. Comparisons of the SNPs identified by whole genome sequence (WGS) and Sanger sequencing.

Supplemental Table S2A. By haplotype

			SNP coordinate																
**Sample**	**Sequencin**	**#Clones**	**387752**	**387868**	**387951**	**387990**	**388003**	**388009**	**388049**	**388068**	**388091**	**388102**	**388104**	**388107**	**388109**	**388126**	**388149**	**388336**	**388342**
BEL	WGS		0/477	414/0	0/375	0/328	331/1	0/282	459/2	477/0	533/0	0/436	454/0	0/447	0/448	488/0	0/398	0/424	438/0
	Sanger	12/12	T		G	T		T				A		T	C		C	A	
C08	WGS		42/156	200/34	15/173	0/177	165/22	25/147	205/8	198/5	202/12	208/2	206/0	201/2	153/51	190/5	176/23	202/7	201/0
	Sanger	34/34	T		G	T		T											
C127	WGS		796/10	791/13	583/19	1/423	456/18	433/22	15/303	18/205	14/202	14/172	200/0	13/175	4/190	18/311	501/9	13/818	16/805
	Sanger	12/12				T			C	T	T	A		T	C	A		A	C
M08	WGS		485/0	109/349	78/385	0/410	65/363	65/302	395/50	378/34	371/25	366/28	401/2	365/29	0/379	356/36	51/340	390/108	517/0
	Sanger	30/36		T	G	T	C	T							C		C		
		6/36				T			C	T	T	A		T	C	A		A	
M19	WGS		0/215	258/0	38/241	30/205	239/0	29/179	259/0	261/0	181/103	244/40	129/146	228/45	34/243	275/0	69/140	294/2	296/0
	Sanger	50/91	T		G	T		T					G		C		C		
		8/91	T		G	T		T											
		14/91	T		G	T		T			T	A		T	C		C		
		19/91	T								T				C				

Supplemental Table S2B. By Reference Allele Frequency (in %)

**Sample**	**Sequencing**	SNP coordinate																
		**387752**	**387868**	**387951**	**387990**	**388003**	**388009**	**388049**	**388068**	**388091**	**388102**	**388104**	**388107**	**388109**	**388126**	**388149**	**388336**	**388342**
BEL	Sanger	100	0	100	100	0	100	0	0	0	100	0	100	100	0	100	100	0
	WGS	100	0	100	100	0	100	0	0	0	100	0	100	100	0	100	100	0
C08	Sanger	100	0	100	100	0	100	0	0	0	0	0	0	0	0	0	0	0
	WGS	79	15	92	100	12	85	4	2	6	1	0	1	25	3	12	3	0
C127	Sanger	0	0	0	100	0	0	100	100	92	100	0	100	100	100	0	100	100
	WGS	1	2	3	100	4	5	95	92	94	92	0	93	98	95	2	98	98
M08	Sanger	0	83	83	100	78	83	17	17	17	17	0	17	100	17	83	17	0
	WGS	0	76	83	100	85	82	11	8	6	7	0	7	100	9	87	22	0
M19	Sanger	99	0	79	79	0	78	0	2	35	15	55	15	91	0	70	0	0
	WGS	100	0	86	87	0	86	0	0	36	14	53	16	88	0	67	1	0
